# The Character of Queen Elizabeth

**Published:** 1922-02

**Authors:** 


### THE REVIEW OF THE MONTH.

### THE CHARACTER OF QUEEN ELIZABETH.

"NO scandal about Queen Elizabeth," says Sneer in "The Critic." And the quotation might well serve as a motto for this volume.* The author makes no secret of his admiration for the great Tudor monarch; he would, in this respect, have satisfied even Charles Kingsley. The word admiration is, indeed, not strong enough. For he regards Elizabeth as having been raised up by Divine Providence for the express purpose of founding the British Empire. He does not boggle at this position, does not just hint at it, but states it in round terms. To him, as to the City Missionary in "No. 5, John Street," it is clear that English, if not the sole language of Heaven, is the one most in use there. At the present day, this somewhat Victorian attitude is pathetic and touching.

But the book is a fascinating one. It possesses historical value and medical interest. To the readers of this Review, the latter aspect has, probably, the greater importance.

The usual view of Queen Elizabeth regards her as a woman of robust health and of Amazonian physique, one who never ailed, who was never tired by the longest day's hunting or evening's dancing, one who, but that she lived in the sixteenth, and not in the twentieth century, would have held her own at hockey, would have surpassed Mlle. Lenglen at lawn tennis, and might even have commanded her fleet in person, and given a hand at squaring the main-yard or serving at a gun. We confess that it was thus that we had envisaged the Queen.

But this conventional view is, as so often happens, quite wrong. The author has collected a vast amount of contemporary evidence to show that Queen Elizabeth had been sickly from her childhood, that she had a number of acute illnesses, and that her general health was that of a "chronic invalid." Nor does the author present this simply as his own conclusion. He has empanelled a jury of medical experts, to whom he has presented his evidence. The names of the jury (the late Sir William Osler, Sir Clifford Allbutt, Sir Arthur Keith, Mr. Alban Doran, and Dr. J. A. Howard, of Norwood) inspire confidence, and no one of them will be challenged "as he comes to the book to be sworn." On the general question of the Queen's chronic ill-health the jury present a unanimous verdict. And we may regard this part of the author's case as proved.

What was the cause of this almost continuous sickness? There is fairly definite evidence that the Queen suffered from nephritis, probably due to scarlatinal infection in early girlhood. There is a history of *two* attacks of smallpox, and it is suggested that one of these may really have been chicken-pox. It is quite likely, however, that it may have been measles, for this disease was not properly differentiated from smallpox until the time of Sydenham in the next century.

But another, and a much more interesting question arises. Did Elizabeth suffer from any pre-natal infection? The author suggests that she did so suffer. He holds that there is much reason to think that Henry VIII. had contracted syphilis, and that he infected his children through their mothers. This opinion has, of course, been enunciated by previous authors. Whether we hold that syphilis was introduced into Europe by the sailors of Columbus, on their return from the West Indies, or whether we take the view that it had been previously known, there is no doubt that a widespread epidemic of syphilis occurred after the siege of Naples in 1494. This is accounted for on either view. Even had the disease been already known, there would be a reinforcement of the virus, just as the syphilitic manifestations in this country, which had for years been growing milder, suddenly acquired fresh virulence under the influence of the new strains introduced during the war from France. We learn from Erasmus that the disease was both prevalent and virulent in England early in the sixteenth century. Henry is known to have had a somewhat lurid youth, and his middle age cannot be accurately painted in dull colours. And it is very probable that he did not escape infection with the lues venerea. Did he infect his children? There is evidence that Edward VI. was so infected, perhaps some evidence in the same direction as regards Mary. But with Elizabeth the position is not so clear. There is nothing in the evidence which points very definitely to this infection. The interesting contemporary portraits of the Queen, six in number, which are reproduced in this volume, show no very marked facial characteristics of congenital syphilis. The jury are not convinced of the overwhelming weight of the evidence on this point. Their verdict here is "not proven." We agree.

What of Elizabeth's mental health? That she was a learned woman we knew already. And the author gives a description of her many linguistic and other accomplishments, which were acquired so early in her life that the modern reader, accustomed to the methods of Froebel and Montessori, stands amazed. In Tudor days, probably very little attempt was made to educate the majority of girls (or boys). Those in whose cases the attempt was made must have had a bad time. So large a list of accomplishments, acquired so early, can only have been achieved at the cost of ruthless suppression of the primitive childish instincts. The "pleasure principle" must have been brought into early and violent opposition to the "reality principle," to use the language of Freud. But, in Elizabeth's case, there is much more to consider. As the author points out, her whole early life was passed under a thick cloud. Her father having divorced his first wife, and pronounced her offspring to be illegitimate, had beheaded Elizabeth's mother on a charge of incest, had pronounced his marriage with her to have been null and void *ab initio*, and Elizabeth herself to be illegitimate. He had married Jane Seymour in what must, even then, have been

* "The Private Character of Queen Elizabeth." By Frederick Chamberlin, LL.B., M.R.I., F.R.H.S., F.S.A., F.R.G.S., F.R.A.S. (John Lane; 18s. net.)

**The Review of the Month—(*cont.*).**

regarded as indecent haste. Much of these happenings would have been concealed from the little girl. We may suppose that those who spoke of them would have been in danger of the rack, the axe, or the quartering knife. But the child must have seen early that there was something gravely amiss with her family life. Her natural inquiries would be met with answers known to be evasive or false. Need we look further for the cause of a "mental conflict," the effects of which upon Elizabeth's mind and character must have been of a most serious nature? Much of Elizabeth's medical history, as set out in this volume, points strongly to the existence of neurotic symptoms, which would be directly due to this repressed mental conflict. May it not be that many of the strange inconsistencies in her conduct which have so puzzled historians, and, above all, her remarkable sex manifestations, have the clue to their solution in this mental conflict? We may well be grateful to Mr. Chamberlin for gathering so laboriously the contemporary "physical" medical evidence. But what would we not give for accurate information as to her "psychic" state? How valuable would it have been, for instance, could some of the Queen's dreams have been analysed!

And if Elizabeth's childhood was a sad and a strenuous one, her girlhood and early womanhood were stormy in the extreme. Imprisoned by her sister, and with her life, at times, in imminent danger on account of the rash attempts at rebellion on the part of her adherents, it would have been strange had she not been embittered. "Princes" quite apart, we may suspect that her motto had become, "Put not your trust in any child of man."

We have placed most stress upon the Queen's environment. But her heredity must not be overlooked. Neither Henry VIII. nor "the fairest daughter of all the Boleyns" was, in any way, an ideal parent. Perhaps, as a nation, we may have cause for congratulation that our present ruling House is so far removed from that "sturdy English stock." It may have been well that the Tudors left no very direct descendants to occupy the throne of these realms.

We have given most of our space to these considerations, because we imagine that the medical parts of this book are those which our readers will peruse with most interest. But Mr. Chamberlin's object has not been to produce a medical chronicle of Elizabeth's health. That, to him, is a purely secondary matter, interesting only in so far as it bears on his main thesis. His great aim has been to free his heroine from the aspersions which have been cast on her moral character. He labours, perhaps unnecessarily, the point that Lingard must be considered as a prejudiced authority. But, quite apart from Lingard, there can be no doubt that Elizabeth has often been regarded as a woman of grossly immoral sexual life. Mr. Chamberlin owns that this had been his own impression. He has seen reason to alter his view. And he is very anxious that others should do so. This is the main objective at which he aims.

He sums up all the evidence which tends to indicate that Elizabeth had immoral relations with Leicester, Hatton, Essex, and Raleigh. He extenuates nothing. He gives us all the evidence which can be drawn from contemporary sources. No other evidence is, of course, worth anything. He then, judicially, places before the reader all the evidence, of the same contemporary kind, which can be urged on the other side. He makes no secret of his view that the verdict on Elizabeth's moral character should be one of "not guilty." But, here, the jury consists of the author's readers; the author, himself, is in the position of the judge who sums up the cause.

We may mention that Sir Arthur Keith describes Elizabeth as a sexless individual. There seems to be no strong evidence for this statement. The Queen's medical history, and her physical conformation, as indicated by the portraits, appear to be against this theory. It is far more likely that her somewhat peculiar relations with the male sex were occasioned by a repressed sex complex.

Mr. Chamberlin puts forward the ingenious theory that the scandals which were rife in her time were put abroad by the express authority of Elizabeth herself. In Mr. Chamberlin's opinion, the Queen's object was to prevent the marriage projects, which were constantly being formed by various foreign princes, from reaching a point at which she would have been obliged to give a definite decision, affirmative or negative. He suggests that it was her policy to keep a number of suitors dangling after her, and not, by bestowing her hand upon one, to offend the others. Readers will judge for themselves how far the author has proved his case in this respect. But if he has done so, then we agree with him in saying that no ruler ever served her land more faithfully. She, on this theory, deliberately sacrificed her reputation for what she regarded as her country's good.

The book deals but briefly with the interesting subject of the Queen's attitude to the rival religions. It is admitted that she conformed to the observances of the "old" religion during the reign of her sister, with the intention of preserving part of her liberty, possibly of saving her life. Such action was, no doubt, prudent and statesmanlike. But it was not the action of a zealot, and it would not have commended itself to the ardent religionists on either side.

Incidentally, the book is designed to displace Burghley from the position, as the Queen's chief adviser, which he has held so long, and, as the author would have it, so undeservedly. Mr. Chamberlin elevates Leicester to a much higher pinnacle of fame. If the Cecil family should happen to read the book, they will not be too well pleased with some of the author's comments upon their famous progenitor, or by his caustic comments upon the opinions expressed by one of their living representatives.

The book is splendidly printed, and there is an excellent index. In addition to the portraits, several interesting specimens of handwriting are reproduced. We can congratulate Mr. Chamberlin on having produced a most readable, interesting and ingenious volume. And we can promise all readers thereof much gratification in perusing it.

